# Intelligent Analysis of Abnormal Vehicle Behavior Based on a Digital Twin

**DOI:** 10.1007/s12204-021-2348-7

**Published:** 2021-10-28

**Authors:** Lin Li, Zeyu Hu, Xubo Yang

**Affiliations:** 1 Shanghai International Automobile City (Group) Co., Ltd., Shanghai, 201805 China; 2 School of Software, School of Electronic Information and Electrical Engineering, Shanghai Jiao Tong University, Shanghai, 200240 China

**Keywords:** digital twin, deep learning, vehicle detection, abnormal behavior, TP 391.4, A

## Abstract

Analyzing a vehicle’s abnormal behavior in surveillance videos is a challenging field, mainly due to the wide variety of anomaly cases and the complexity of surveillance videos. In this study, a novel intelligent vehicle behavior analysis framework based on a digital twin is proposed. First, detecting vehicles based on deep learning is implemented, and Kalman filtering and feature matching are used to track vehicles. Subsequently, the tracked vehicle is mapped to a digital-twin virtual scene developed in the Unity game engine, and each vehicle’s behavior is tested according to the customized detection conditions set up in the scene. The stored behavior data can be used to reconstruct the scene again in Unity for a secondary analysis. The experimental results using real videos from traffic cameras illustrate that the detection rate of the proposed framework is close to that of the state-of-the-art abnormal event detection systems. In addition, the implementation and analysis process show the usability, generalization, and effectiveness of the proposed framework.

## 0 Introduction

In recent years, with the continuous development of the economy, people's living standards continue to improve. An increasing number of vehicles are on the road, and correspondingly, traffic congestion and accidents occur more frequently. The main cause of traffic congestion and accidents is abnormal vehicle behavior; therefore, it is important to analyze vehicle behavior intelligently. In terms of vehicle behavior detection, traditional detection tools mainly include magnetic induction coils, infrared rays, and ultrasonics[1]. Although these traffic detection tools play a certain role in alleviating traffic flow and pressure, their application is limited by high maintenance costs and complex installation of hardware equipment. In recent years, with the continuous development of computer vision technology, vehicle behavior detection based on video monitoring has gradually been applied[2].

Vehicle behavior detection based on video surveillance mainly includes target detection, target tracking, and trajectory motion analysis. Traditional image processing methods are used in target detection, which can only detect the moving vehicle target and cannot classify the vehicle type. Moreover, the detection speed is slow, the accuracy is low, and the result is easily affected by the external environment. The tracking effect with methods such as the Kalman filter, particle filter and CamShift after detection is poor because of the low detection accuracy, slow detection speed, and frequent missing detection in the vehicle target detection phase, which also affects the track motion analysis.

A digital twin is applied to the vehicle behavior analysis system to overcome these problems. At present, the wave of information technology represented by cloud computing, big data, artificial intelligence, virtual reality, autonomous driving, and other new technologies has swept the world, also affecting urban planning, construction, and development[3]. The urban virtual image constructed in bit space superimposed on the urban physical space will greatly change the appearance of the city, reshape the urban infrastructure, and create a new form of urban development with the combination of virtual reality and twin interaction. A more intelligent new city will be created with a faster network and more intelligent computing. As an important part of urban management, intelligent digitalization is the future development direction of urban transportation systems. A digital twin is a mapping relationship between the physical space and the virtual space, which blends virtual reality and intelligent control[4-5]. Digital-twin transportation is a widespread application of digital-twin technology at the transportation level. Intelligent analysis of vehicle behavior is one of the applications of digital twins.

In this study, a novel vehicle behavior analysis framework based on a digital twin is proposed. The framework mainly consists of five parts as follows: video capturing, YOLOv5-based vehicle detection[6], tracking, mapping of the relationship between the real scene and the digital-twin virtual scene, and analysis of the vehicle's behavior.

The input to the system can be a real-time signal from a surveillance camera or a local video. Through the deep learning algorithm YOLOv5 and the tracking algorithm, we can obtain the vehicle's trajectory and its 2D coordinates in the video coordinate system. The 2D coordinates can be transferred to 3D coordinates according to the mapping relationship between the surveillance video and virtual scene created in the Unity engine scene. Afterwards, each detected vehicle is 3D reconstructed in the virtual scene. The reconstructed vehicle model can interact with the behavior detection condition set up in the virtual scene. For example, through a collision zone, the system can detect if the vehicle changes lanes. The detected information is structurally stored in the database, so that it can be called again later to reproduce the behavior of the vehicle and conduct secondary observation and analysis.

This study aims to analyze vehicle behavior based on digital-twin technology. Our contributions are summarized as follows: detection of a vehicle's color, type, and position using a deep learning model based on YOLOv5; a set of virtual scene construction processes and mapping schemes between the real scene and the digital-twin virtual scene; a methodology to design a vehicle behavior detection strategy based on regulations and its implementation in the Unity engine; a structured vehicle behavior storage database for reconstructing the vehicle's behavior, which also stores more vehicle data than the surveillance video.

## 1 Vehicle Detection and Tracking Algorithm

Vehicle detection module is the first stage of our vehicle behavior analysis framework (Fig. 1).

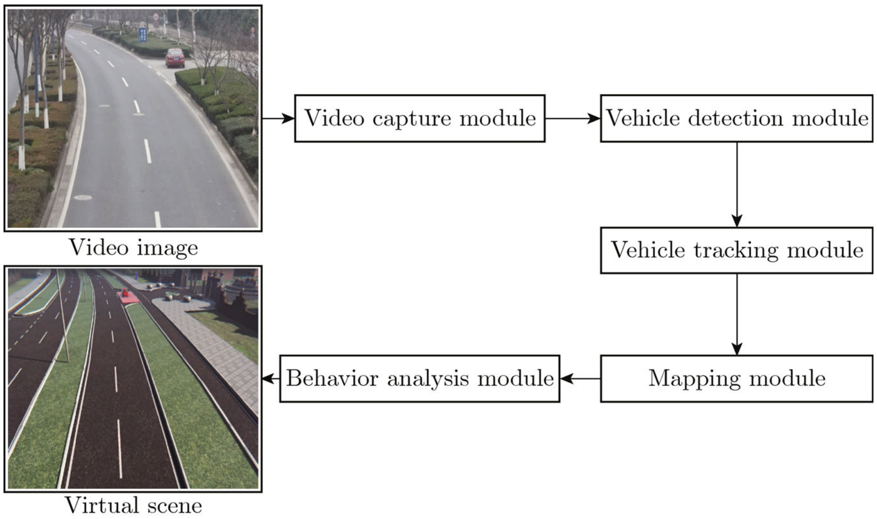

Fig. 1 System framework

### 1.1 Deep Learning Algorithm YOLOv5

As the latest member of the YOLO target detection family, YOLOv5 adds several important modules. Among them are mosaic data enhancement and adaptive anchor frame calculation in the input, focus and cross stage partial network (CSP)[7] in the backbone, and feature pyramid network (FPN)[8] and pixel aggregation network (PAN)[9] in the neck, which improves the detection accuracy and speed. It can be said that YOLOv5 is currently one of the most advanced detection models.

According to the different depths of the network, YOLOv5 has four different models: s, m, l, and x. The YOLOv5s's network structure is illustrated in Fig. 2. In the CSP1 structure, the YOLOv5s model has the minimum model x, i.e., (1, 3, 3), as shown in Fig. 2, which means that the number of residual components is minimal. In the CSP2 structure, YOLOv5s also has the minimum model x, i.e., (1, 1, 1, 1), as shown in Fig. 2, which indicates that the number of CBL blocks is minimal. YOLOv5m's model x is twice as much as YOLOv5s's model x, so the model x in CSP1 is (2, 6, 6) and the model x in CSP2 is (2, 2, 2, 2). YOLOv5l's model x is triple that of YOLOv5s's model x, and YOLOv5x's model x is quadruple that of YOLOv5s's model x.

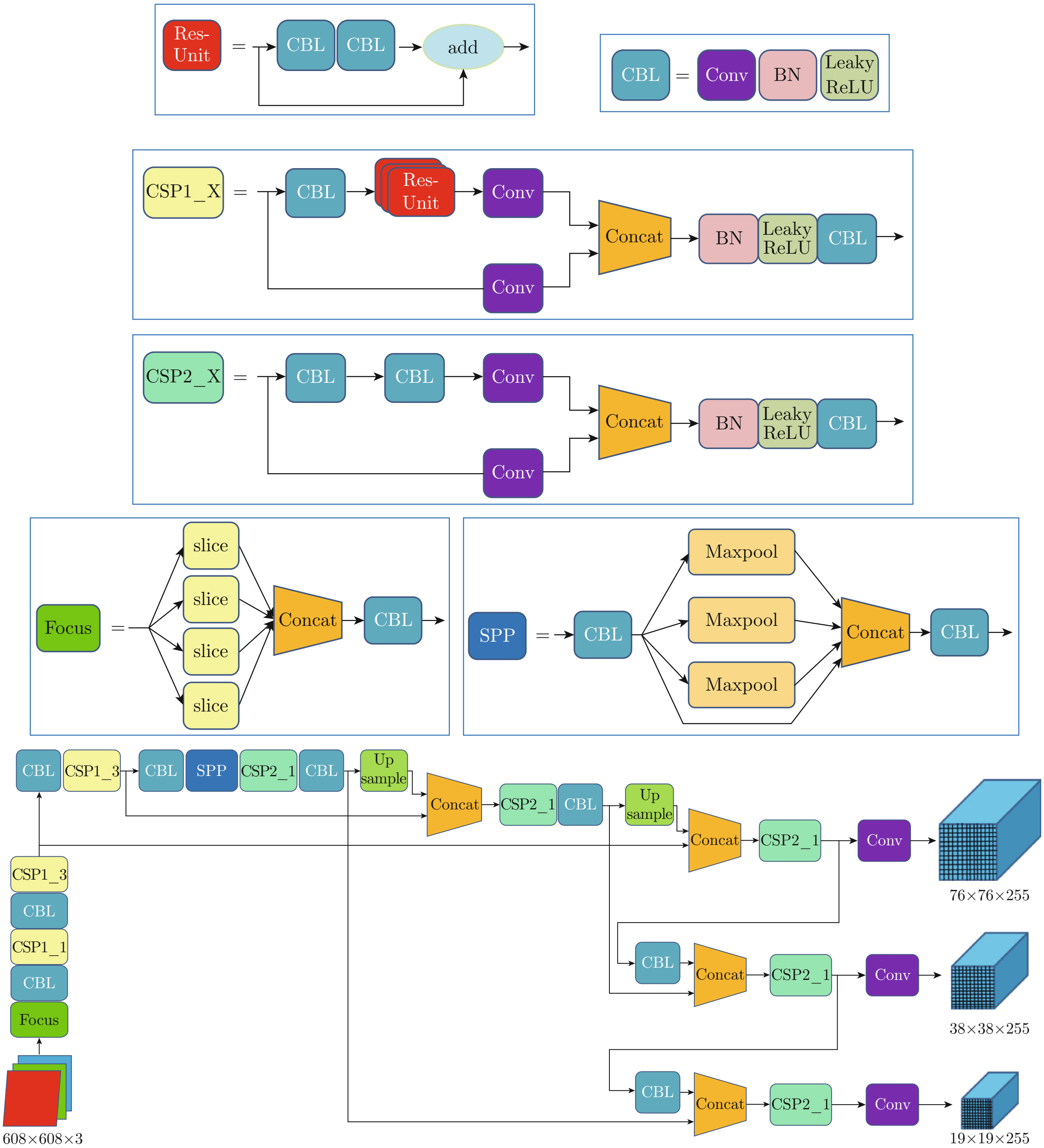

Fig. 2 YOLOv5s's network structure①

YOLOv5s's computing speed is the fastest, while its Average Precision (AP) score is the lowest. The other three models have higher AP scores, but their time consumption on a single frame also decreases from the model s to the model x. Their concrete performance is shown in Table 1. Comprehensively considering the performance and accuracy, in this project, we chose YOLOv5m as our detection model. In Table 1, $a_{\text{test}}$ represents the model's AP result on the COCO 2017 test-dev dataset[10], $a_{\text{val}}$ represents the model's AP

①This image is licensed by JiangDaBai.

result on the COCO 2017 validation dataset, $s_{\mathrm{GPU}}$ measures how many frames are calculated per second while the model is running on the n1-standard-16 V100 (16 vCPUs Intel Haswell, 60 GB memory, NVIDIA V100 GPU), and $t_{\mathrm{GPU}}$ measures how long it takes to compute a frame while the model is running on the n1-standard-16 V100. Finally, Params represents the model's size.

**Table 1 Performance of YOLOv5's different models on n1-standard-16 V100**

| Model | $a_{\mathrm{test}}$/% | $a_{\mathrm{val}}$/% | $s_{\mathrm{GPU}}$/ (frame·s$^{-1}$) | $t_{\mathrm{GPU}}$/ ms | Parmas× $10^{-6}$ |
|---|---|---|---|---|---|
| YOLOv5s | 36.6 | 36.6 | 476 | 2.1 | 7.5 |
| YOLOv5m | 43.4 | 43.4 | 333 | 3.0 | 21.8 |
| YOLOv5l | 46.7 | 46.6 | 256 | 3.9 | 47.8 |
| YOLOv5x | 48.4 | 48.4 | 164 | 6.1 | 89.0 |

### 1.2 UA-DETRAC Dataset and YOLOv5 Model Retraining

For detecting the vehicle's color and type, we chose the UA-DETRAC dataset[11] as our training set. UA-DETRAC is a challenging real-world multi-object detection and multi-object tracking benchmark. The dataset consists of 10 h of videos captured with a Cannon EOS 550D camera at 24 different locations in Beijing and Tianjin in China. The videos were recorded at 25 frames per second (FPS), with a resolution of 960 pixel × 540 pixel. There are more than 140 thousand frames in the UA-DETRAC dataset and 8 250 vehicles that are manually annotated, leading to a total of $1.21 \times 10^6$ labeled bounding boxes of objects, as shown in Fig. 3.

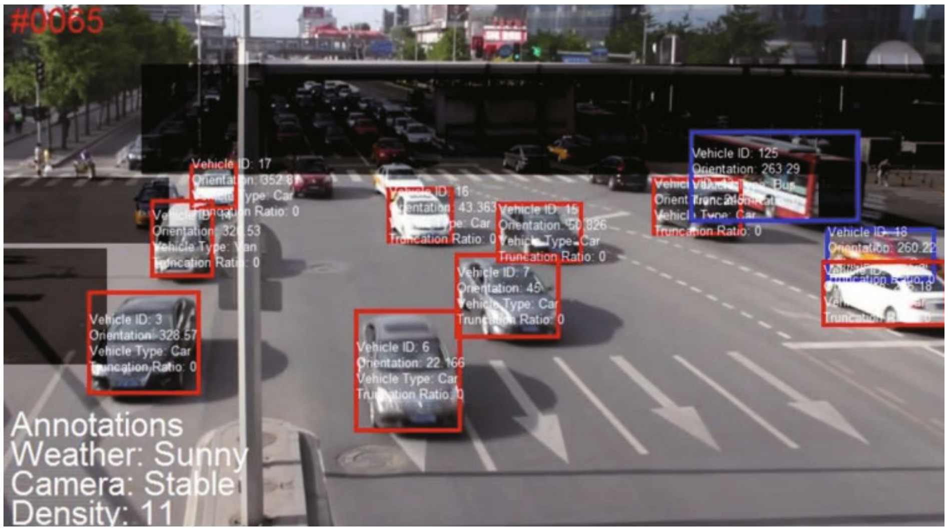

Fig. 3 Example of UA-DETRAC benchmark②

Each grid cell of the original YOLOv5 model predicts three boxes. Each box needs five basic parameters, $x$, $y$, $w$, $h$, and the confidence. Here, ($x$, $y$) are the coordinates of the center point of the box, $w$ represents the width of the box, and $h$ represents the height of the box. With 80 classes of probabilities on the COCO data set, each grid outputs a 255-dimensional tensor, $(80 + 5) \times 3 = 255$. To adapt the new UA-DETRAC dataset, we changed the YOLOv5 model. The vehicle data of UA-DETRAC has two attributes (13 types + 12 colors), and each grid outputs a 90-dimensional tensor, $(13 + 12 + 5) \times 3 = 90$. The detection results are shown in Fig. 4.

According to the vehicle's position information, color, type, and features obtained by vehicle detection, the vehicle can be tracked. The vehicle tracking algorithm uses the Kalman filter algorithm[12] to predict the updated trajectory. The Kalman filter is a type of

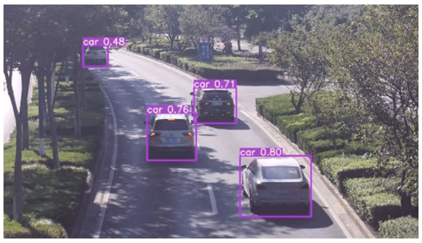

(a) Before retraining

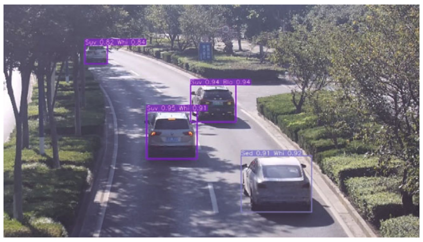

(b) After retraining

Fig. 4 Detection results before and after retraining YOLOv5m

②This image is licensed under CC BY-NC-SA 3.0.

recursive estimation. Through the estimated value of the previous state and the observed value of the current state, the estimated value of the current state can be calculated. After the prediction and update of the Kalman filter, the position information of the vehicle predicted at the current time can be obtained. Afterwards, a cost matrix is calculated by the intersection and union ratio of the bounding box between each detected vehicle target and each predicted vehicle target. By combining the cosine distance of the color and type feature vector obtained by the YOLOv5 feature between the detected vehicle and the predicted vehicle, the Hungarian algorithm[2] is used to match, and the vehicle target tracking can be realized.

## 2 Virtual Scene Establishment and the 2D to 3D Mapping Relationship

### 2.1 Virtual Scene Establishment

To achieve a digital twin, we should build a 1 : 1 virtual scene compared to real city scenes. For large-scale scenarios, traditional manual construction cannot meet the efficiency requirements. Our solution is an innovative and efficient scene construction tool chain and production process, which integrates a high-definition map, data collection, photogrammetry[13] and process generation technology, as shown in Fig. 5.

The highest accuracy is required for the road part. We needed to reconstruct the road model based on the accurate information from the high-definition map or the actual collected data. A high-definition map contains the geometry, type, indication, and other information related to the road and traffic, which is usually stored in vector form. A high-definition map is an important cornerstone of digital twins.

Photogrammetry is an efficient method for reconstructing a model with rich details by using camera shooting and computer vision. It is a very effective construction method for ancillary facilities and buildings around the roads. It can achieve highly consistent results with real objects and a realistic and natural rendering effect.

For large-area terrain and non-major buildings, we used a process modeling method to generate virtual scene automatically based on geographic information, which not only ensures consistency with the real scene, but also greatly improves the efficiency, and makes full use of data information to further improve the efficiency of scene data storage and rendering.

Taking Fig. 6 as an example, from geographic information system (GIS) data or other high-precision map information, we can obtain the geometry, type,

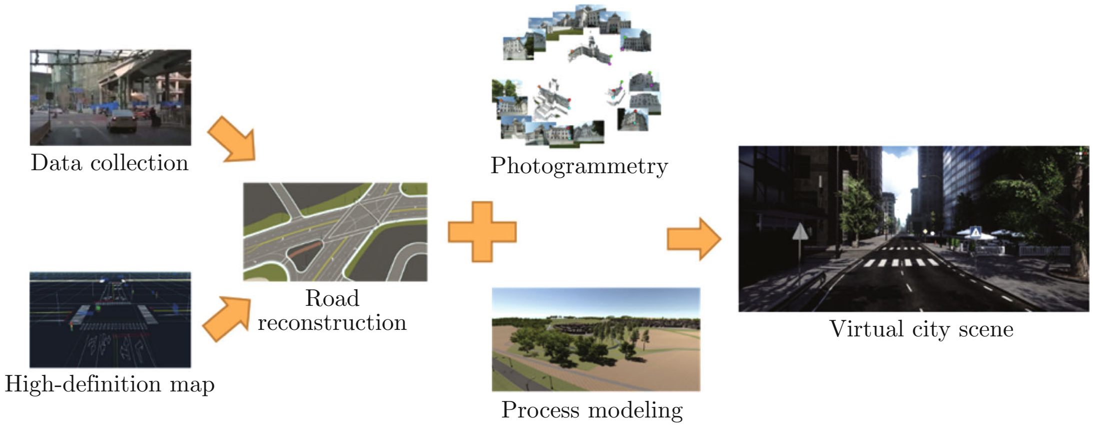

Fig. 5 Virtual scene construction tool chain

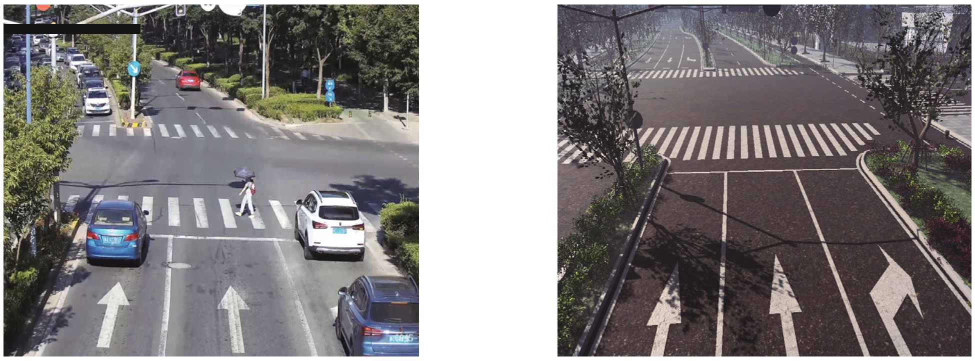

(a) Grid video image (b) Partial corresponding grid nodes in the virtual scene

Fig. 6 Virtual scene in Unity at 1 : 1 scale indication, and other information related to the road. The geometry data helped us build a rough road model in Unity. The type of data determines the road texture. The indication information and the image of the surveillance camera can help us to deploy road signs, direction signs, traffic lights, and other traffic instructions. We then added more details, such as buildings nearby, bushes, or trees by photogrammetry.

### 2.2 The 2D to 3D Mapping Relationship

We have obtained the vehicles' 2D coordinates in the video, and a 3D virtual scene corresponding to a real city scene in a video. Now, we need to establish a mapping relationship from 2D coordinates in video to 3D coordinates in a virtual scene.

First, we draw grids on the video image and take the points of the grid nodes in the unity 3D scene, as shown in Fig. 7. Then, we generate the corresponding relationship between the video coordinates $(u, v)$ of each grid node and the 3D coordinates of the position in a Unity 3D scene. Finally, each identified vehicle's 2D coordinates can be transferred to 3D coordinates using the bilinear interpolation method. The advantages of this method are high precision, quantifiable error control, and no dependency on the camera's internal and external parameters that are difficult to obtain accurately.

The formula of bilinear interpolation for each point in the grid is

$$f(u,v) = [1-u \quad u]\begin{bmatrix} f(0,0) & f(0,1) \\ f(1,0) & f(1,1) \end{bmatrix}\begin{bmatrix} 1-v \\ v \end{bmatrix}, \quad (1)$$

where $u,\ v \in [0,1]$ represent the distance ratios of the $X$ and $Y$ axes from the origin, respectively. According to the corresponding relation of the grid nodes, the formula can be rewritten as

$$g(f(u,v)) = \\ [1-u \quad u]\begin{bmatrix} g(f(0,0)) & g(f(0,1)) \\ g(f(1,0)) & g(f(1,1)) \end{bmatrix}\begin{bmatrix} 1-v \\ v \end{bmatrix}, \quad (2)$$

where $g(f(u,v))$ denotes the corresponding 3D coordinates of $f(u,v)$ in the virtual scene, and $f(0,0)$, $f(0,1)$, $f(1,0)$, and $f(1,1)$ are the coordinates of the four nearest grid nodes.

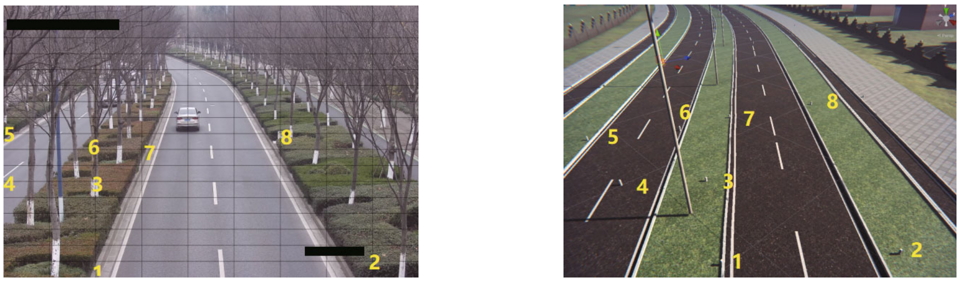

(a) Grid video image (b) Partial corresponding grid nodes in the virtual scene

Fig. 7 Illustration of 2D to 3D mapping relationship

## 3 Recognizing the Vehicle's Behavior in the Unity Virtual Scene

### 3.1 Unity Trigger Mechanism

Unity[14] released their first version of Unity in 2005, targeting only the OS X platform; since then, Unity has developed and upgraded the support to more than 25 platforms, including virtual and augmented reality. Unity is a multi-platform integrated game development tool that allows users to easily create interactive content such as 3D video games, architectural visualization, and real-time 3D animation by manipulating 2D or 3D objects and attaching several components to them. It has a powerful rendering engine, a PhysX physics engine, and a collision detection engine.

The most important feature of Unity that we used to judge a vehicle's behavior is the collision detection trigger mechanism. It is used to trigger three kinds of unity game object interactive events, as shown in Fig. 8.

When a game object such as a vehicle model with a collision bounding box enters the trigger area, the on_trigger_enter() function attached to the trigger area is called. When it is in the trigger area, the on_trigger_stay() function is called once per frame. When it leaves the trigger area, the on_trigger_exit() function is called.

### 3.2 Different Case Detection

Common vehicle behaviors in traffic systems include running red lights, legal diversions, illegal diversions, legal parking, illegal parking[15-17], etc.

Different detection strategies are designed for different cases[18].

**Diversion Cases** The main types of lane lines are as follows: yellow double solid lines, yellow single solid lines, white solid lines, yellow dotted lines, and white dotted lines. The main difference between them is that vehicles can cross dotted lines and not solid lines[19]. The diversion detection zone is designed as a strip that can cover the lane line area in the virtual scene, as

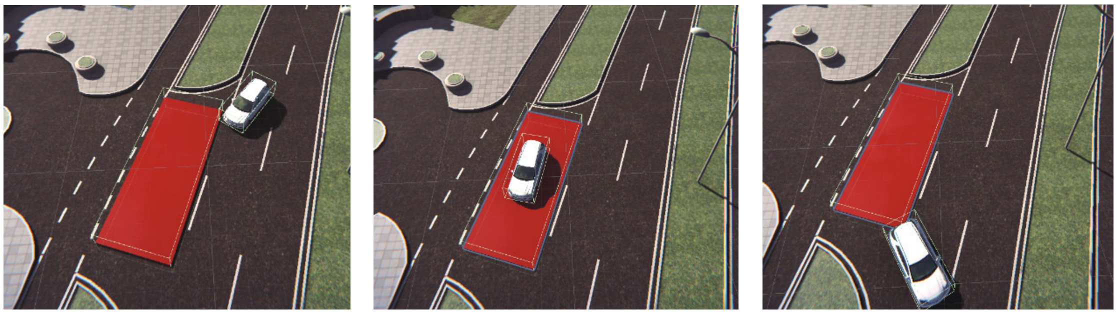

(a) Trigger enter (b) Trigger stay (c) Trigger exit

Fig. 8 Three kinds of trigger events shown in Fig. 9. The green zone, which can detect legal diversions, is set up for sections with dotted lines. The red zone, which can detect illegal diversions, is set up for sections with solid lines. When the vehicle object in the virtual scene's collision box enters the trigger zone, the corresponding case is detected and its detailed information, including case name, occurrence location, vehicle type, vehicle color, vehicle license number, case start date, and case end date, which are accurate to the millisecond, is recorded in the database.

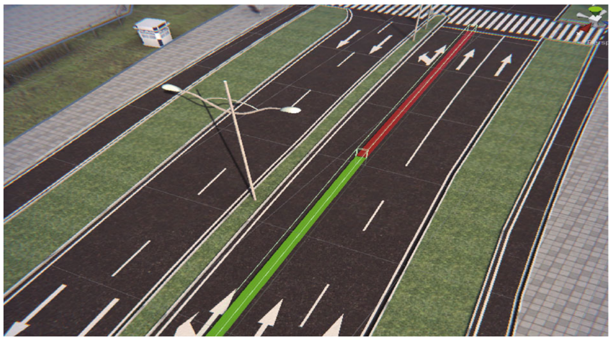

Fig. 9 Diversion case detection zone in a virtual scene

**Parking Cases** The common illegal parking areas include sections with no stopping signs and markings, sections with isolation facilities between motor vehicle lanes and non-motor vehicle lanes and sidewalks, pedestrian crossings and construction sections, railway crossings, sharp bends, narrow roads less than 4 m in width, bridges, steep slopes, tunnels, and sections within 50 m of the above places[20]. The parking detection zone is designed as a rectangle that can cover the illegal parking area in the virtual scene, as shown in Fig. 10. Unlike diversion case detection, parking detection needs to measure the duration of parking. Therefore, we set a timer in the on_trigger_stay() void, which is called once per frame when a vehicle is in the parking detection zone. Case information is also recorded in the database.

**Cases Involving Running Red Light** Running a red light refers to the behavior of a motor vehicle that

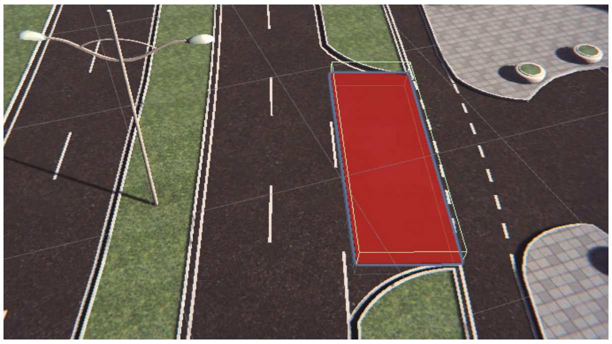

Fig. 10 Parking case detection zone in a virtual scene crosses the stop line and continues driving when the red light is on and continuing into the intersection is not allowed[21-22]. The red light detection zone is designed as a rectangle that can cover the illegal parking area in the virtual scene, as shown in Fig. 11. Compared with parking and diversion case detection, running a red light is more complicated. Real-time signal data are required to determine whether a vehicle has run a red light. Therefore, an intersection always needs two or more detection zones for driving straight and turning situations. Case information is also recorded in the database.

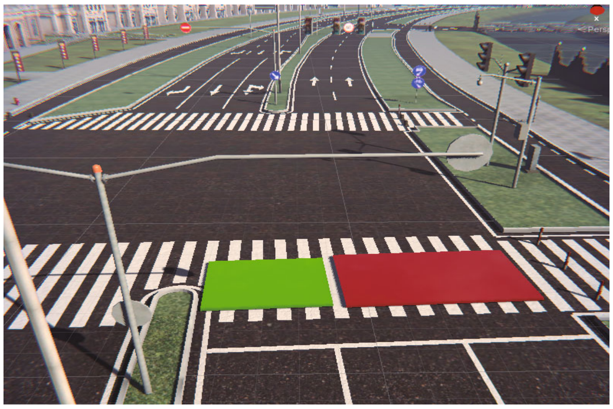

Fig. 11 Running a red light detection zone in a virtual scene

It is easy to arrange the detection zones based on Unity in a virtual scene by setting several prefabs. A prefab is a resource type, a reusable game object stored in the project view. Consequently, when the game requires many repeated objects, such as the detection zones in our system, the prefab can play an important role. It has the following characteristics. First, the prefab can be put into multiple scenes. They can also be placed multiple times in the same scene. This means that when a diversion case detection zone prefab is created, we can replicate it and put the replicas in all the positions that correspond to road lines in reality. Second, no matter how many instances exist in the project, it is only necessary to change the prefab, and all prefab instances will change accordingly. Therefore, if the detection logic has been changed, we do not need to modify every detection zone object, and we only need to modify the prefab. In addition, every object can be specially modified, and changes to only one object will not affect the other objects. Consequently, for some special locations, the vehicle behavior detection strategy can be customized.

### 3.3 Vehicle's Behavior Reappearance in Unity

Using the previous methods, we can detect a vehicle's behavior and obtain detailed data on this behavior. These data are structured and stored in the database. The structure of the database is shown in Fig. 12. As seen in Fig. 12(a), "name" refers to the behavior's name, such as illegal parking and illegal diversion. Further, "vid" refers to the ID of the camera that captures the vehicle. The attribute "tid" is the vehicle's tracking ID in one camera. The attribute "gid" is the vehicle's unique global ID in the entire system. In addition, "startdate" and "enddate" refer to the vehicle's behavior's start time and end time, respectively. They are all accurate to the microsecond. The attributes ($x$, $y$, $z$) are the position coordinates of the location in the virtual scene. And "kind", "color", and "license" are the feature information of the vehicle. According to the global ID of the vehicle, more detailed vehicle movement data can be queried in the origin data table, as shown in Fig. 12(b). Most attributes in this table have the same meaning as in the behavior table. Attribute "date" refers to the time point of this piece of data, and it is real-time. Therefore, the system can reproduce the vehicle's behavior in the Unity virtual scene.

This application is also one of the advantages of digital twins. The corresponding 3D virtual scene provides a platform to reproduce the scene, such as replaying surveillance video. It can also meet the requirements of multi-angle, partial suspension, and overall control when the vehicle behavior needs to be observed again. In addition to these advantages, through this method we can store longer monitoring data than by storing videos. Usually limited by insufficient hard disk space, the road camera stores video from half a month to a month. If structured data are stored, the storage time can be significantly improved.

| Behavior |
| --- |
| name: STRING |
| vid: INT |
| tid: INT |
| gid: INT |
| startdate: BIGINT |
| enddate: BIGINT |
| $x$: FLOAT |
| $y$: FLOAT |
| $z$: FLOAT |
| kind: STRING |
| color: STRING |
| license: STRING |

(a) Vehicle behavior data table

| Origin |
| --- |
| vid: INT |
| tid: INT |
| gid: INT |
| date: BIGINT |
| $x$: FLOAT |
| $y$: FLOAT |
| $z$: FLOAT |
| kind: STRING |
| color: STRING |
| license: STRING |

(b) Vehicle real-time position table

Fig. 12 Structure of the database

## 4 Analysis of Results

To obtain better detection results, we collected 20 surveillance videos from the 1 000 m section of the Boyuan Road in Shanghai. Each video lasted approximately 5 min, and a total of 80 000 samples were obtained. All vehicles in each frame are labeled, including the type, color, and bounding box. After the training samples were constructed, the PyTorch framework was used to train on the NVIDIA RTX2080 GPU. Combined with UA-DETRAC data, 200 epochs were trained with a 640 pixel × 640 pixel image size, which is suitable for the vehicle size in the 1 920 pixel × 1 080 pixel surveillance video. After the training, we obtained a YOLOv5m model that can detect the vehicle's type, color, and bounding box. The detection results are shown in Fig. 4.

In the evaluation of the algorithm, the AP $a$, mean Average Precision (mAP) $\bar{a}$, and FPS $s$ were selected as evaluation indexes. The AP can reflect the performance of the model in detecting specific types of vehicles. By averaging the AP values of all vehicle types, mAP can not only reflect the average performance of the model for all vehicle types, but also avoid the problem that some types are more extreme and weaken the performance of other types. The FPS reflects the number of frames that can be processed per second, known as the running speed of the algorithm. The performance indices are listed in Table 2. It has a higher recognition rate for several common vehicles in the training set. In future work, data with rarer vehicle types will be added for data enhancement.

**Table 2 Test performance index**

| Type | $a$/% | $\bar{a}$/% | $s$/(frame·s$^{-1}$) |
|---|---|---|---|
| Sedan | 96.81 | | |
| SUV | 96.45 | | |
| Truck | 95.27 | 94.31 | 58 |
| Van | 94.24 | | |
| Bus | 93.55 | | |
| Others | 89.55 | | |

Then, by using the tracking algorithm and the 2D to 3D model, the vehicle can be transferred into the digital-twin virtual scene. In the virtual scene, we have set up the detection zones according to the actual situation. If the condition is satisfied, the vehicle's behavior is detected and recorded in the database, as shown in Fig. 13. Figure 13(a) is the digital twin of Fig. 13(b). The green strip zone is the diversion detection zone, and it is triggered by the white vehicle. So, this legal diversion case has been detected, and its data has also been stored. Figure 13(c) is the digital twin of Fig. 13(d). The red rectangle zone is the parking detection zone, and it is triggered by the red vehicle. So, this illegal parking case has been detected, and its data has also been stored.

By querying the database, we can reproduce the vehicle behavior. As Fig. 14 shows, Fig. 14(a) is the first time the vehicle's behavior was detected. Figure 14(b) shows the reproduced scene. Users can observe the entire scene from different perspectives. Figure 14(c) shows that the system can reproduce the vehicle's route before and after the occurrence of the incident to obtain a more comprehensive grasp of vehicle behavior.

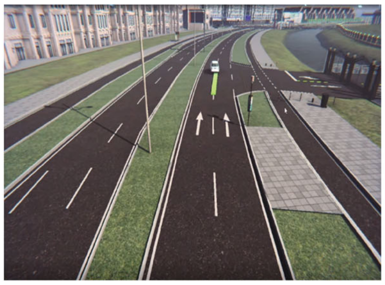

(a) Legal diversion detected in virtual scene

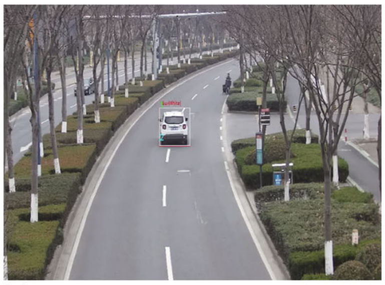

(b) Corresponding surveillance video

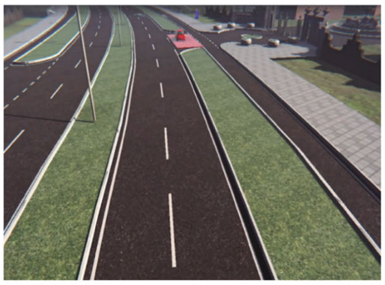

(c) Illegal parking case detected in virtual scene

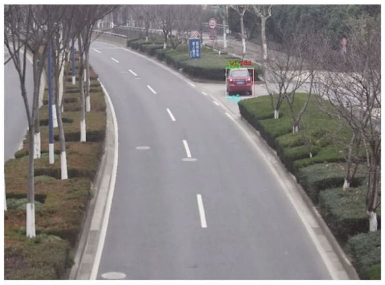

(d) Corresponding surveillance video

Fig. 13 Intelligent detection of vehicle behavior based on digital twin

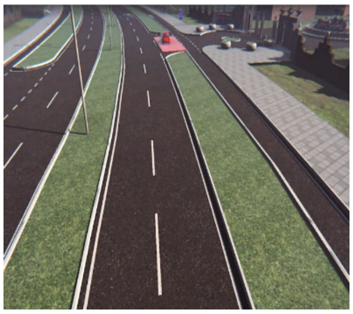

(a) Original scene

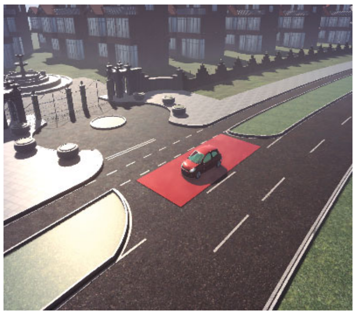

(b) Reproduced scene from a different perspective

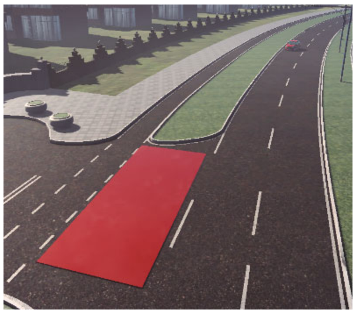

(c) Vehicle's route

Fig. 14 Vehicle's Behavior Scene Reappearance in Unity

For quantitative results, the evaluation is based on the vehicle behavior detection performance, measured by the F1-score[23]. A true-positive (TP) detection is considered to occur when the predicted behavior is exactly the true behavior of the vehicle. The time difference between the recorded behavior and the actual occurrence time must be no more than 5 s. A false-positive (FP) is a predicted anomaly that is not a TP for a given anomaly. Finally, a false negative (FN) is a true behavior that is not correctly predicted. Hence, the F1-score is measured by

$$\mathrm{F1}=\frac{2\mathrm{TP}}{2\mathrm{TP}+\mathrm{FP}+\mathrm{FN}}. \tag{3}$$

The test was conducted on 20 surveillance videos on a selected road. Each video takes a different 40-min period from the previous training, including straight roads and intersections. We compared our behavior detection method with three state-of-the-art methods from studies by Xie, Wang, and Zheng, as shown in Table 3. These three methods are most relevant to ours, and our method provides some kind of improvement over theirs. It can be seen that our method's F1-score reaches the second highest in the diversion case and the highest in the parking case. There is not enough data for running the red light; therefore, this behavior is not included in the test. However, because of the similar detection principle, we can infer that the accuracy will be at the same level.

**Table 3 Comparison of the abnormal behavior detection effects**

| Study | F1-score | | F1-score | |
|---|---|---|---|---|
| | Legal diversion | Illegal diversion | Legal parking | Illegal parking |
| Ref. [20] | 95.4 | 95.7 | 97.5 | 97.2 |
| Ref. [22] | 94.2 | 94.4 | 96.4 | 96.8 |
| Ref. [23] | 96.8 | 97.8 | 98.0 | 98.2 |
| Ours | 96.5 | 97.4 | 98.2 | 98.4 |

The F1-score for parking is higher than the other F1-scores. One of the reasons is that the parking detection zone is always close to the camera, so that the vehicle detection is stable. This is also the reason why the F1-score of illegal diversions is higher than the F1-score of legal diversions. The end of the camera screen is often a dotted line. Another reason why the F1-score for parking is higher, is that the parking detection zone is always a separate area off the road so that there is no other vehicle to block the candidate vehicle.

## 5 Conclusion

To realize the intelligent analysis of vehicle behavior, we propose a novel vehicle behavior analysis framework based on a digital twin. A deep learning algorithm based on YOLOv5m is used to detect vehicles. Then, target tracking is performed using a Kalman filter and feature matching to obtain the vehicle trajectory. Through the 2D to 3D model, the identified vehicles can be mapped to the virtual scene built in advance. This process is also known as a digital twin. The detection trigger conditions are designed according to real traffic laws and regulations, which are intuitive for users. Each behavior only needs to preset a detection area prefab, which can be easily arranged in all regions of interest. Experiments demonstrate that this framework performs well in detecting a range of abnormal behaviors. In addition, this framework can reproduce the vehicle's behavior in the Unity virtual scene by calling the structured vehicle behavior data that exists in the database.

However, when the vehicle targets have a large area of occlusion, stay away from the camera, or are vehicle types with a small sample set, missed or false detections still exist, which will affect the tracking effect of the vehicle and even the accuracy of the analysis results of vehicle behaviors. Therefore, one of the focus areas of future research is expanding the dataset and performing more accurate detection when the target is occluded. Currently, there is no recognition of pedestrians or non-motor vehicles. More efforts will be devoted to detecting them and achieving the corresponding digital twin. Combined with the input of pedestrian and non-motor vehicles, the system can detect vehicle behaviors related to pedestrian and non-motor vehicles.

**Open Access** This article is licensed under a Creative Commons Attribution 4.0 International License, which permits use, sharing, adaptation, distribution and reproduction in any medium or format, as long as you give appropriate credit to the original author(s) and the source, provide a link to the Creative Commons licence, and indicate if changes were made. The images or other third party material in this article are included in the article's Creative Commons licence, unless indicated otherwise in a credit line to the material. If material is not included in the article's Creative Commons licence and your intended use is not permitted by statutory regulation or exceeds the permitted use, you will need to obtain permission directly from the copyright holder. To view a copy of this licence, visit http://creativecommons.org/licenses/by/4.0/.
